# Ethnobotanical research in Cava de’ Tirreni area, Southern Italy

**DOI:** 10.1186/s13002-019-0330-3

**Published:** 2019-10-17

**Authors:** Mattia Mautone, Laura De Martino, Vincenzo De Feo

**Affiliations:** Dipartimento di Farmacia, Università degli Studi di Salerno, Via Giovanni Paolo II, 132, 84084 Fisciano, Salerno, Italy

**Keywords:** Traditional medicine, Cava de’ Tirreni, Ethnobotany, Ethnopharmacology, Traditional uses

## Abstract

**Background:**

To best of our knowledge, this is the first quantitative ethnobotanical study with the aim of documenting the local knowledge and practices of using plants for curing diseases in the Cava de’ Tirreni area, Salerno Province, Campania Region, Italy. The present ethnobotanical field study, carried out during 2016–2017, documents the local uses of 119 plant species for medicinal, food and domestic purposes.

**Methods:**

Ethnobotanical data were documented from 70 informants: field data were collected and information on the uses of plants was gathered through semi-structured and structured interviews with persons who still retain traditional ethnobotanical knowledge. Documented data were evaluated using the quantitative ethnobotanical index of use value (UV).

**Results:**

Overall, the informants native of the area were interviewed and 277 use-reports have been recorded. The scientific names, local names, plant part used, preparation and administration processes are reported and compared with practices in other Southern Italian regions. In total, 101 species are documented as medicinal, 36 as food or food aromatizer, 29 for domestic and handicraft uses, 10 in veterinary medicine. More or less 64% of all species have more uses and over half of the food plants (23 species) are also used for medicinal purposes.

**Conclusions:**

The comparison of the documented species and their uses with ethnobotanical literature of other Italian regions reveals that the traditional plant knowledge in this area shows strong similarities with adjacent Southern Italian areas. Some of the recorded species and administration processes however seem to be unique for the zone.

## Background

Since ancient times medicinal plants belonged to the history of the man who tried to insert them in the context in which he lived. The ecology of Mediterranean area, inhabited for millennia, has been strongly influenced by human–nature relationships, increasing the variability of landscapes [1]. Ethnobotanical studies show that traditional plant knowledge still survives in different areas of the Mediterranean region, particularly among seniors [2, 3]: in this area, numerous plants are widespread and used by people in different, complex, and evolving ways. But the comprehension of these processes is still basic [4] and the ethnobotanical research goes on to find novel or unusual employments of also well-known medicinal plants [4]: in this way, the ethnobotanical use of a plant becomes a continuous developing process, influenced by environmental and cultural factors.

The aims of this study are to deepen the ethnobotanical knowledge of the Cava de’ Tirreni area (Campania, Southern Italy), for saving and comprehending this precious information. Specifically, the finalities of our research are to (i) improve and conserve knowledge about the traditional plant uses in the Cava de’ Tirreni area and (ii) explore the gathered data, comparing them with ones present in ethnobotanical bibliography of other Southern Italian regions, to find possible linkages with other nearby areas.

## Methods

### Study area

**Fig. 1:**
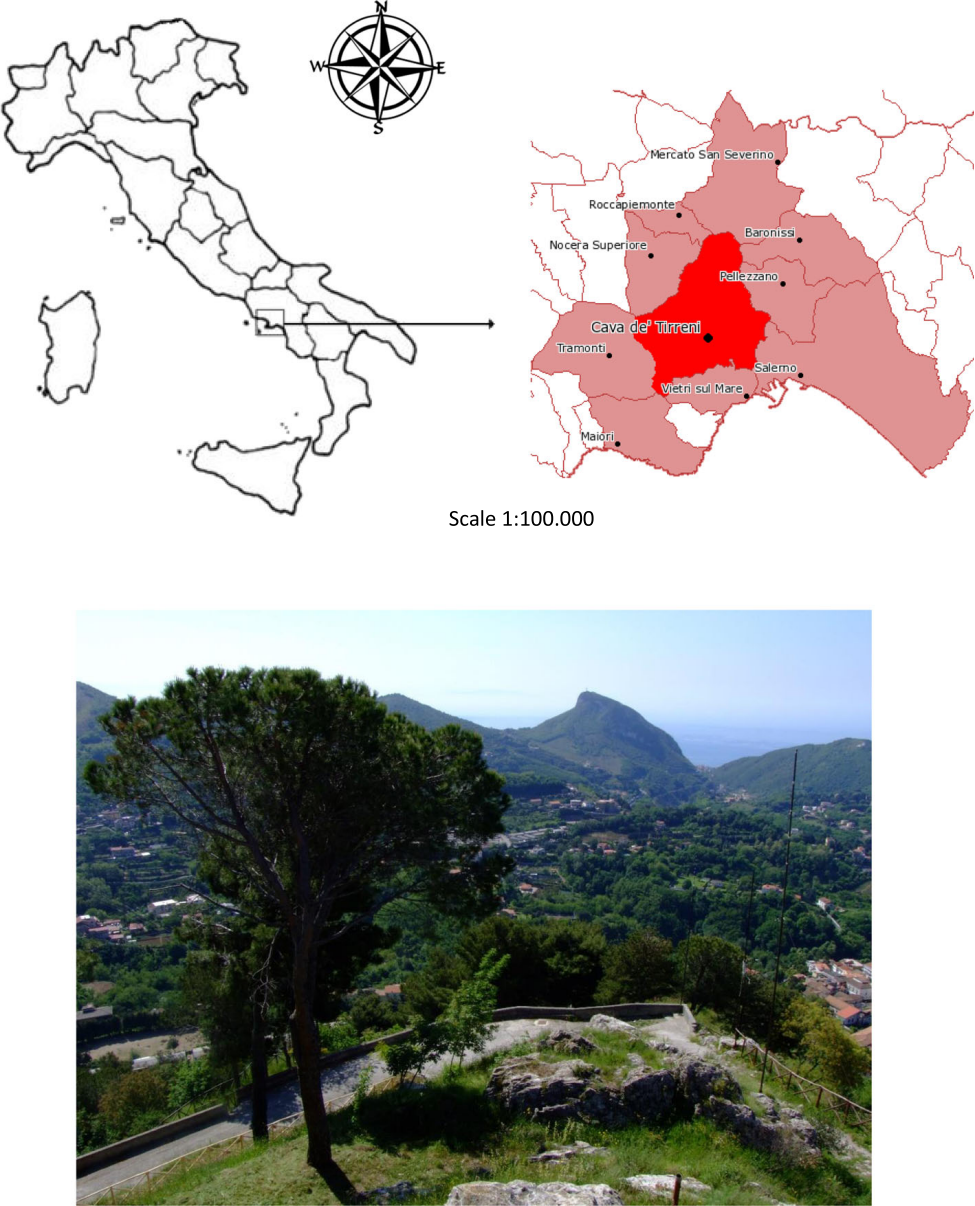
Cava de’ Tirreni, Campania, Southern Italy

The Cava de’ Tirreni area (Campania, Southern Italy, Fig. 1) is surrounded by two vast mountain ranges, in Northern and Southern directions, at a latitude of 40° and 40′ north and a longitude of 32° and 20′ East, (200 m a.s.l.). This area spread over 35 km^2^, at the Northern borders of Salerno Province. We focused our research in this area because of its isolation and its economy, which is still partially based on small-scale agricultural and pastoral activities. We believe that this mountainous locality represents a potential interesting area for conducting studies on traditional ethnobotanical knowledge.

The area has a Mediterranean climate, with hot summers and wet winters. The coldest months are January and February with temperatures of 7.9 °C and 8.6 °C, while the hottest months are July and August with temperatures of 31.6 °C and 31.2 °C. The annual rainfall average is 1025 mm for 106 rainy annual days [5].

The area of Cava de’ Tirreni has been populated since ancient times, with a large part of its surface characterized by cultivations. Within cultivated species, the most important horticultural plants are belonging mainly to Solanaceae, Fabaceae, and Brassicaceae families and fruit plants belonging to Rosaceae. *Morus* spp., *Ficus carica* L., *Punica granatum* L., and *Diospyros kaki* Thunb. *J. regia* and *Corylus avellana* L. are also widespread, as well as *Citrus limon* (L.) Burm. f. Osbeck, *Citrus aurantium* var. *dulcis* L., and *Citrus reticulata* Blanco. Also, the cultivation of *Vitis* L., with different varieties, is diffused.

Besides cultivated fields, the area is also characterized by natural vegetation with a high level of biodiversity: this reflects both the presence of different substrates, such as limestone and thick soils of volcanic origin, and the presence of numerous microclimates, due to the fact that the area includes altitudinal bands ranging from 200 to 1000 m above sea level and exposed slopes in all directions [5].

The natural vegetation comprises a mosaic of woodlands and shrubland vegetation (maquis and garrigue). Typical woody species are *Alnus cordata* (Loisel.) Desf., *Acer opalus subsp. obtusatum* (Waldst. and Kit. ex Willd.) Gams, *Quercus pubescens* Willd., *Olea europoea* L., and *Ceratonia siliqua* L in the woodlands and *Myrtus communis* L., *Pistacia lentiscus* L., *Rosmarinus officinalis* L., *Helichrysum italicum* (Roth) G. Don, *Juniperus phoenicea* L., in the shrubland vegetation.

### Ethnobotanical methods

Field data were collected, in several time intervals, during the period April 2016–October 2017 and ethnobotanical information on the applications of studied plants were gathered through semi-structured and structured interviews with people who actually know local traditions [1].

The selection of people was made at random among the oldest persons who still conserve traditional knowledge about medicinal plants [6].

In the beginning part of the field study, people were invited to name all medicinal and useful plants and remedies utilized in the past. Other accurate information were registered in a second phase, through structured interviews with the aim to complete a suitable questionnaire [7] (Additional file 1).

The interviewed people were asked to provide a fresh specimen of each plant cited for systematic identification, to call it in the local dialect (Salernitan dialect of Italian language) and to show its properties, ways of administrations, and employments (in human and veterinary medicine, as human food and animal feed, in the agricultural, domestic, or handcraft fields). A fresh sample of each plant was shown to the informants to avoid a misidentification of the species [8]. In some cases, it has been asked to interviewees to show the objects named during the conversation, as crates, brooms, hand tools, and sticks. If a plant was cited without having any herbal specimen, the informant was invited to go to the field and show the named species. A careful control analysis has been made after collecting the data and identifying the species, to avoid of including non-traditional information, for example originated from books or audiovisual materials.

The informants interviewed were 70 (29 men, 41 women), whose ages ranged from 50 to 95 years, and belonged to families more representative of the area. Most of the interviewees (59) were aged over 60, of whom 40 were between 60 and 69, 18 between 70 and 90, and 1 was over 90 years old. Among the informants, 25 were farmers; the others were employed in the construction, restaurants, and sheep-farming. They all were born and inhabited in the studied area for many years. The informants know that the information they furnished will be published.

The methodology employed in this study uses the qualitative data of classical ethnobotanical-systematic research on plants, and the numerical quantitative data of consensus, following the guides for ethnobotanical studies [7–10].

The results of the present work are compared to ethnobotanical data of contiguous zones, to confirm the medicinal uses or report some differences [5, 6, 11–29].

Voucher herbarium specimens were compressed, classified, dried and stored in the Herbarium of the Medical Botany Chair at the University of Salerno. The volumes of Flora di Pignatti [30] were used for the classification and nomenclature of plants: finally, all the names were updated using the site http://www.theplantlist.org/.

### Data analysis

We utilized the use value to calculate the most frequently used plants. The use value [31] was calculated to determine the relative importance of a species according to the following formula:

$$ \mathrm{UV}=\mathrm{U}/\mathrm{N} $$

where, UV is the use value of the species, U is the number of informants, and N is the total number of informants.

## Results and discussion

The list of the useful and medicinal plants and their uses are presented in Table 1. For each plant, the following information are provided: botanical name and family, voucher specimen number, local name, part used and prescription, and use value. The research led to the identification of 119 plants belonging to 52 families, of which the more widely represented are Asteraceae (16), Lamiaceae (11), Brassicaceae (6), Solanaceae (6), Umbelliferae (5). This survey revealed that the majority of species have been reported in ethnobotanical literature: for few others, the cited uses are present only in the traditional knowledge of this area. The plant uses can be divided into four main categories: plants for (i) medicinal use (101 species, 197 uses), (ii) veterinary use, including plants used as feed (10 species, 13 uses), (iii) human food and food aromatizer (36 species, 37 uses), and (iv) domestic and handicrafts use (29 species, 30 uses).

**Table 1:** Plants traditionally used in Cava de’ Tirreni

Family/species (Herbarium number)	Salernitan name	Parts used	Uses recorded	UV
Aceraceae				
*Acer campestre* L. (Mattia 094)	Nocefragola	Wood	Dom: the wood is used to make tool handles, toys, “ciaramella” (typical musical instrument) and “ziccaro” (bird-call).	0.171
			Med.: the decoction is used in the treatment of amenorrhea and as an abortive.	0.042
Adoxaceae				
*Sambucus nigra* L. (Mattia 011)	Savùco	Bark	Med.: boiled in water, it is used as a lenitive for burns; mixed with olive oil or beeswax, it is claimed to act as a cicatrizer.	0.528
		Leaves	Med.: crushed, they are applied as a lenitive for burned skin.	0.271
			Med.: a poultice, prepared also with leaves of *Parietaria officinalis* and *Vincetoxicum hirundinaria*, is used topically against leg edemas.	0.228
			Food: fresh leaves are eaten cooked with eggs.	0.657
		Inflorescences	Med.: a decoction is employed as a febrifuge.	0.457
			Med.: an infusion is drunk to treat joint inflammations.	0.342
			Med.: An infusion is claimed to cure the female sterility.	0.228
			Med.: used in the preparation of the decoction called “o’ ricotto”.	0.557
		Fruits	Food: Used for typical jams.	0.400
Araceae				
*Arum italicum* Miller (Mattia 043)	Pane ‘e serpe	Rhizome	Med.: topically, it used as a skin decongestant.	0.029
Araliaceae				
*Hedera helix* L. (Mattia 116)	Ellera	Fresh leaves	Med.: boiled until the leaves become a gel and this is used topically as an anti-rheumatic.	0.185
Asclepiadaceae				
*Vincetoxicum hirundinaria* Medik (Mattia 108)	Fetenti	Fresh leaves	Med.: a poultice in olive oil with beeswax is claimed to be an anti-inflammatory in case of traumas.	0.214
			Med: a decoction is used as a gargle for toothache.	0.100
			Dom.: a water maceration with *Urtica dioica* leaves is sprayed on the vegetables to send away insects.	0.185
Aspleniaceae				
*Ceterach officinarum* DC. (Mattia 182)	Spaccaprete	Aerial parts	Med: a decoction is used as an expectorant.	0.314
Boraginaceae				
*Borago officinalis* L. (Mattia 186)	Verraccine; Vurràina	Aerial parts	Food: cooked in salads or with eggs.	0.557
			Med.: a decoction is used as a diuretic.	0.414
*Symphytum tuberosum* L. (Mattia 047)	Cugliunciello	Roots	Med.: the minced roots are applied externally to resolve contusions and wounds.	0.328
Cactaceae				
*Opuntia ficus-indica* (L.) Mill.	Figurine	Branches	Med.: the inner gel is used as lenitive for skin.	0.271
Cannabaceae				
*Cannabis sativa* L. (Mattia 034)	Canapa	Branches	Dom.: mixed with eggs, the fibers were used to make bandages.	0.614
			Dom.: they are used as textile fibers for rope production.	0.728
Capparaceae				
*Capparis spinosa* L. (Mattia 144)	Chiapparo	Buds	Food: used to aromatize foods.	0.771
Caryophyllaceae				
*Saponaria officinalis* L. (Mattia 055)	Erva saponara	Leaves	Dom.: fresh leaves are used to clean hands, especially after tobacco manufacturing.	0.557
Compositae				
*Achillea millefolium* L. (Mattia 103)	Troneto	Flowering tops	Food: used for preparation of liqueurs.	0.285
			Dom.: to make brooms.	0.228
			Med.: the inhalation of its decoction is claimed to possess vermifuge activity.	0.114
			Med.: used in the preparation of the decoction called “o’ ricotto”.	0.557
*Artemisia absinthium* L. (Mattia 078)	Nascienzo	Fresh leaves	Med.: a decoction is claimed to be an anti-diabetic.	0.785
			Food: used for preparation of liqueurs.	0.528
*Bellis perennis* L. (Mattia 005)	Margherita sarvatica	Flower heads	Dom.: a maceration is used to prepare a cosmetic scented water.	0.185
			Med.: a decoction is claimed to be febrifuge.	0.200
*Cichorium intybus* L. (Mattia 063)	Cicoria	Aerial parts	Food: cooked in preparation of “minestra maritata”.	0.685
			Med.: a decoction is used as a laxative.	0.442
			Med.: a decoction is claimed to be a liver depurative.	0.385
*Centaurea benedicta* (L.) L. (Mattia 046)	Cardogna	Aerial parts	Feed: they are used as a special feed for donkeys.	0.271
*Condrilla juncea* L. (Mattia 163)	Lattarole	Aerial parts	Food: cooked in preparation of “minestra maritata”.	0.514
*Crepis vesicaria* L. (Mattia 187)	Lattarole	Aerial parts	Food: cooked in preparation of “minestra maritata”.	0.514
*Cynara cardunculus ssp. scolymus* (L.) Hayek (Mattia 009)	Carcioffa	Leaves	Med.: a decoction is used in treatment of liver disease.	0.314
*Helminthotheca echioides* (L.) Holub (Mattia 098)	Lattarole	Aerial parts	Food: cooked in preparation of “minestra maritata”.	0.514
*Lactuca sativa* L. (Mattia 114)	Nzalata	Leaves	Med.: boiled leaves are used topically in case of toothache.	0.557
*Matricaria chamomilla* L. (Mattia 133)	Camumirra	Flowering heads	Med.: an infusion with *Laurus nobilis* leaves is used topically for edemas.	0.328
			Med.: a poultice is used topically as an eye anti-inflammatory.	0.628
			Med.: An infusion, taken orally, is claimed to be a sedative.	0.714
			Med.: a poultice is applied externally in case of hematomas and traumas.	0.642
			Med.: used in the preparation of the decoction called “o’ ricotto”.	0.557
*Reichardia picroides* (L.) Roth (Mattia 054)	Lattecielle	Leaves	Food: cooked in preparation of “minestra maritata”.	0.371
*Silybum marianum* (L.) Gaertn (Mattia 113)	Cardone	Flowering heads	Food: cooked in preparation of “minestra maritata”.	0.514
*Sonchus oleraceus* (L.) L. (Mattia 171)	Stracciacannarone	Aerial parts	Food: cooked in preparation of “minestra maritata”.	0.514
*Tanacetum balsamita* L. (Mattia 132)	Erva da’ madonna	Aerial parts	Med.: used in the preparation of the decoction called “o’ ricotto”.	0.471
*Taraxacum campylodes* G.E. Haglund. (Mattia 158)	Cicoria sarvatica	Leaves	Food: uncooked in salads or cooked in preparation of “minestra maritata”.	0.514
Convolvulaceae				
*Calystegia sepium* (L.) R. Br. (Mattia 056)	Campanelle	Whole plant	Med.: a decoction is used as a hypotensive.	0.114
Corylaceae				
*Ostrya carpinifolia* Scop. (Mattia 228)	Carpino	Leaves	Med.: used in the preparation of the decoction called “o’ ricotto”.	0.514
Cruciferae				
*Armoracia rusticana* Gaertner, B.Mey, and Scherb. (Mattia 004)		Leaves	Dom.: leaves are smoked.	0.057
*Brassica oleracea* L. (Mattia 041)	Caveleciore	Leaves	Med.: a decoction is used externally to treat furuncles.	0.342
			Med.: internally, a decoction is claimed to be a depurative.	0.142
*Capsella bursa-pastoris* (L.) Medik. (Mattia 102)	Zeppolelle sarvatiche	Leaves	Food: cooked in preparation of “minestra maritata”.	0.514
			Med.: fresh leaves are eaten as an antispasmodic in case of colic.	0.185
*Diplotaxis tenuifolia* (L.) DC. (Mattia 077)	Rucola	Leaves	Med.: cooked leaves are eaten with olive oil and lemon juice as an antispasmodic in case of colic.	0.214
			Med.: a decoction is used as an ophthalmic anti-inflammatory.	0.085
			Med.: an infusion is claimed to be a men aphrodisiac.	0.500
*Lobularia maritima* (L.) Desv. (Mattia 095)	Ciurilli ianchi	Flowering tops	Med.: a decoction is used as a febrifuge.	0.014
			Med.: a decoction is employed as a peripheral vasodilator.	0.057
			Med.: a decoction is taken orally as a prostatic anti-inflammatory.	0.157
*Nasturtium officinale* R.Br. (Mattia 146)		Leaves	Food: in salads or cooked in preparation of “minestra maritata”.	0.514
Cucurbitaceae				
*Cucurbita pepo* L. (Mattia 105)	Cocuzza	Fruits	Dom.: dry fruits were used as seeds containers.	0.371
		Seeds	Med.: they are eaten as a vermifuge.	0.614
			Med.: they are eaten in case of constipation.	0.442
Equisetaceae				
*Equisetum arvense* L. (Mattia 093)	Cola ‘e volpe	Aerial parts	Med.: a decoction is used in treatment of prostate and bladder affections.	0.171
Ericaceae				
*Arbutus unedo* L. (Mattia 231)	Sovera pelosa	Leaves	Med.: a decoction is used internally as an astringent.	0.485
		Fruits	Food: they were eaten fresh or in jams.	0.400
Euphorbiaceae				
*Euphorbia dendroides* L*.* (Mattia 168)	Tutamaglio	Latex	Med.: it was applied topically to treat warts.	0.542
		Whole plant	Dom.: a water macerate is sprayed on fruit-trees to prevent theft.	0.214
*Mercurialis annua* L. (Mattia 124)	Murcuvella	Aerial parts	Med.: an infusion is used as a general tonic.	0.228
			Med.: an infusion is claimed to act as a digestive.	0.114
			Med.: an infusion is employed as a febrifuge.	0.214
Fagaceae				
*Castanea sativa* Mill. (Mattia 096)	Castagno	Seeds	Food: to prepare cakes and pasta.	0.242
			Feed: as a food for pigs.	0.685
		Wood	Dom.: it was used to make vats, barrels, kitchen utensils, baskets, windows, furniture; today it is used as a stake for arbor.	0.471
*Quercus ilex* L. (Mattia 117)	Elece	Leaves and bark	Med.: a decoction with *Urtica dioica* leaves is used in gargles against throat inflammations.	0.385
		Leaves and acorns	Feed: as a food for pigs.	0.685
		Wood	Dom.: it was used to make vats, barrels and domestic tools.	0.557
*Quercus robur* L. (Mattia 127)	Cerza	Leaves and bark	Med.: a decoction with *Urtica dioica* leaves is used in gargles against throat inflammations.	0.357
		Leaves and acorns	Feed: as a food for pigs.	0.685
		Wood	Dom.: it was used to make vats, barrels and domestic tools.	0.471
Graminaceae				
*Arundo donax* L. (Mattia 036)	Canna	Rhizome	Med.: a decoction is used in treatment of gastric affections.	0.228
		Branches	Dom.: to make baskets and musical instruments; as a support for vegetables.	0.557
*Cynodon dactylon* (L.) Pers. (Mattia 104)	Gramigna	Rhizome	Med.: a decoction is employed as an urinary anti-inflammatory and as a diuretic with *Urtica dioica* leaves.	0.442
			Med.: used in the preparation of the decoction called “o’ ricotto”.	0.557
		Whole plant	Med.: an infusion is claimed to be useful in treatment of women infertility.	0.185
*Triticum turgidum* L. (Mattia 045)	Grano	Seeds	Feed.: dirty dishes are washed with bran in hot water; this water was then given to domestic animals to drink.	0.314
*Zea mays* L. (Mattia 089)	Gravurino	Stigmas	Med.: a decoction is used as a diuretic and for treatment of kidney stones.	0.442
Guttiferae				
*Hypericum perforatum* L. (Mattia 031)	Erva di san Giuvanni	Flowering tops	Med.: a decoction is claimed to be a prostate anti-inflammatory.	0.157
			Med.: used in the preparation of the decoction called “o’ ricotto”.	0.428
		Aerial parts	Med.: crushed fresh plants or an olive oil macerate were used as a lenitive and cicatrizer.	0.271
Juglandaceae				
*Juglans regia* L. (Mattia 072)	Noce	Leaves	Med.: a decoction is claimed to be useful in treatment of hyperglycemia.	0.657
			Dom: they are put in bean sacks to keep away insects.	0.242
		Husk	Med.: used in the preparation of the decoction called “o’ ricotto”.	0.514
Labiatae				
*Ajuga reptans* L. (Mattia 066)	Erva d’a’ Maronna	Leaves and flowers	Med.: a decoction is claimed to be useful in treatment of renal diseases.	0.085
*Lavandula angustifolia* Mill. (Mattia 013)	Spigandos	Flowers	Med.: a decoction is employed in treatment of gastro-intestinal diseases.	0.171
			Med.: a decoction is employed in treatment of urinary diseases.	0.142
			Med.: an infusion with *Papaver rhoeas* petals is used as a sedative.	0.328
		Flowering tops	Med.: used in the preparation of the decoction called “o’ ricotto”.	0.557
			Dom: “pupatelle” were prepared and used to wash and to perfume undergarments and to keep away insects.	0.714
*Mentha xpiperita* L. (Mattia 097)	Amenta	Flowers and leaves	Med.: used in the preparation of the decoction called “o’ ricotto”.	0.557
		Leaves	Food: as a main ingredient of a typical food with calf or pork spleen.	0.514
*Mentha* x *rotundifolia* (L.) Huds. (Mattia 121)	Amenta	Flowers and leaves	Med.: used in the preparation of the decoction called “o’ ricotto”.	0.557
		Leaves	Food: as a main ingredient of a typical food with calf or pork spleen.	0.514
*Mentha spicata* L. (Mattia 064)	Amenta	Leaves	Med.: an infusion is claimed to help spleen functionality.	0.142
			Food: as a main ingredient of a typical food with calf or pork spleen.	0.514
		Flowers and leaves	Med.: used in the preparation of the decoction called “o’ ricotto”.	0.557
*Nepeta cataria* L. (Mattia 173)	Nepeta	Flowers and leaves	Med.: used in the preparation of the decoction called “o’ ricotto”.	0.557
		Leaves	Med.: a decoction is used as an antitussive.	0.357
			Dom.: to wash undergarments.	0.228
*Ocimum basilicum* L. (Mattia 022)	Vasenicola	Fresh leaves	Med.: a decoction is employed as a diuretic.	0.142
			Med.: used in the preparation of the decoction called “o’ ricotto”.	0.557
*Origanum vulgare* L. (Mattia 049)	Arecana	Flowering tops	Med.: a decoction is used in treatment of respiratory diseases.	0.471
			Med.: they are applied externally as a lenitive for burns.	0.557
*Rosmarinus officinalis* L. (Mattia 052)	Rosamarina	Aerial parts	Med.: a decoction is considered to act as a general tonic.	0.471
			Med.: used in the preparation of the decoction called “o’ ricotto”.	0.557
*Salvia officinalis* L. (Mattia 153)	Sarvia	Flowers and leaves	Med.: a decoction is claimed to reduce the excessive menstrual flux.	0.100
			Med.: to alleviate gastric pains, a decoction is drunk half an hour after eating an egg albumen.	0.371
			Med.: Crushed fresh leaves are applied on *Herpes zoster* skin lesions.	0.114
			Med.: used in the preparation of the decoction called “o’ ricotto”.	0.557
*Thymus vulgaris* L. (Mattia 030)	Timo Scerapuglia	Flowers and leaves	Med.: a decoction in used in treatment of enteric afflictions and colitis.	0.142
			Med.: vapor inhalation is considered an antitussive and an expectorant.	0.257
			Med.: used in the preparation of the decoction called “o’ ricotto”.	0.557
Lauraceae				
*Laurus nobilis* L. (Mattia 020)	Lauro	Leaves	Food: used as an aromatizer for food and liqueurs.	0.442
			Med.: used in the preparation of the decoction called “o’ ricotto”.	0.557
			Med.: a decoction is used as a digestive.	0.442
			Med.: a decoction is employed as a diuretic.	0.114
Leguminosae				
*Ceratonia siliqua* L. (Mattia 015)	Sciuscella	Seeds	Dom.: in the past, they were used to make necklaces and as a unit of weight.	0.114
		Fruits	Food: as a food for children.	0.228
			Feed: as a food for horses.	0.571
			Med.: the fresh fruit is eaten in case of constipation.	0.157
			Med.: juice was applied topically as on warts.	0.314
*Spartium junceum* L. (Mattia 129)	Janesta	Flowers	Med.: a decoction is considered useful in treatment of diabetes.	0.114
		Leaves	Med.: crushed fresh leaves were applied topically on warts.	0.114
Liliaceae				
*Allium sativum* L. (Mattia 032)	Aglio	Bulbs	Med.: fresh bulbs are applied as decongestant for insect bites.	0.657
			Med.: fresh bulbs are rubbed on corns.	0.557
			Med.: a bulb necklace or vapor inhalations were used against enteric parasites.	0.228
			Vet.: an olive oil macerate is used against chicken diseases.	0.414
*Aloe barbadensis* Mill.		Gel	Med.: applied topically as a skin lenitive.	0.142
*Asparagus acutifolius* L. (Mattia 006)	Spalice	Aerial parts	Food: cooked with pasta or with eggs.	0.414
			Med.: eaten fresh, they are considered to act as a diuretic.	0.457
*Ruscus aculeatus* L. (Mattia 042)	Scacciasurece	Aerial parts	Food: in salads or with eggs.	0.285
			Dom: used to make brooms; to keep out mice	0.614
Malvaceae				
*Althaea cannabina* L. (Mattia 107)	Malvone	Leaves	Med.: fresh crushed leaves were applied as a cicatrizer on wounds.	0.414
*Malva sylvestris* L. (Mattia 065)	Mavca	Leaves and roots	Med.: used in the preparation of the decoction called “o’ ricotto”.	0.557
		Leaves and flowers	Med.: an infusion is claimed to ameliorate blood circulation.	0.071
		Root	Med.: a decoction with a dried fig and apple peel is used as an antitussive.	0.342
Moraceae				
*Ficus carica* L. (Mattia 019)	Fica	Syconia	Med.: a decoction with dried fig leaves and apple peel is used as an antitussive; somebody add walnut hulls, *Malva sylvestris* leaves and *Matricaria chamomilla* heads.	0.357
		Latex	Med.: it is applied on warts.	0.671
		Leaves and dried syconia	Med.: used in the preparation of the decoction called “o’ ricotto”.	0.542
*Morus alba* L. (Mattia 157)	Ceveza janca	Leaves	Med.: a decoction is used as an anti-diabetic.	0.228
			Med.: a decoction is employed as a diuretic.	0.257
*Morus nigra* L. (Mattia 155)	Ceveza nera	Leaves	Med.: a decoction is used as an anti-diabetic.	0.228
			Med.: a decoction is employed as a diuretic.	0.257
Myrtaceae				
*Eucalyptus globulus* Labill. (Mattia 073)	Calipso	Leaves	Med.: vapor inhalation with *Urtica dioca, Cynodon dactylon* roots, *Parietaria officinalis* and lemon leaves are used against sinusitis.	0.514
*Myrtus communis* L. (Mattia 081)	Murtella	Leaves	Med.: an infusion is drunk in case of feet swelling.	0.228
		Leaves and flowers	Med.: a decoction is claimed to ameliorate peripheral circulation.	0.185
			Med.: a decoction is used as an astringent.	0.471
			Med.: used in the preparation of the decoction called “o’ ricotto”.	0.557
		Fruits	Food: used to prepare liqueurs.	0.714
Oleaceae				
*Fraxinus ornus* L. (Mattia 135)	Uorn	Leaves	Med.: used in the preparation of the decoction called “o’ ricotto”.	0.514
		Stem juice	Med.: used as a laxative.	0.385
		Leaves and bark	Med.: a water macerate is used a gastric antispasmodic; the same preparation is claimed to ameliorate liver functions.	0.228
		Bark	Food: a water macerate is used as a refreshing drink.	0.242
			Vet.: a water macerate is used in treatment of “pepitola”, a chicken disease similar to a cold.	0.414
			Feed: used as a food for chicken.	0.414
*Olea europaea* L. (Mattia 154)	Aulivo	Leaves	Med.: a water macerate is used as a hypotensive.	0.271
		Fruits	Med.: fresh fruits are administered to treat hypotension.	0.271
		Wood	Dom.: used to make kitchen utensils and musical instruments (“ciaramella”).	0.185
Papaveraceae				
*Chelidonium majus* L. (Mattia 162)	Papagno sarvatico	Whole plant	Med.: a decoction is claimed to ameliorate liver functions.	0.485
		Latex	Med.: applied topically on warts.	0.742
*Papaver rhoeas* L. (Mattia 003)	Papagno	Flowers/buds	Med.: an infusion is used in treatment of insomnia.	0.671
Plantaginaceae				
*Plantago lanceolata* L. (Mattia 048)	Cincheniervi	Leaves	Med.: crushed and boiled, they are applied to treat furuncles.	0.528
			Med.: crushed, they are applied on contusions and are applied on insect bites.	0.685
			Med.: an infusion is used in treatment of kidney stones.	0.442
			Food: cooked in preparation of “minestra maritata”.	0.514
*Plantago major* L*.* (Mattia 051)	Cincheniervi	Leaves	Med.: crushed and boiled, they are applied to treat furuncles.	0.528
			Med.: crushed, they are applied on contusions and are on insect bites.	0.685
			Med.: an infusion is used in treatment of kidney stones.	0.442
			Food: cooked in preparation of “minestra maritata”.	0.514
Polygonaceae				
*Polygonum aviculare* L. (Mattia 159)	Cientnurehe	Whole plant	Med.: an infusion is considered to be a cholagogue.	0.271
			Med.: used in the preparation of the decoction called “o’ ricotto”.	0.514
			Dom.: boiled with *Foeniculum vulgare* plant, *Laurus nobilis*, *Nepeta cataria*, and lemon leaves, it is used to wash barrels.	0.514
			Med.: an infusion is used to stimulate child appetite.	0.314
Polypodiaceae				
*Polypodium vulgare* L. (Mattia 010)	Filece	Rhizome	Med.: a decoction is used as a vermifuge.	0.271
		Branches	Dom.: used as a carpet where winter apples are placed to mature.	0.342
Portulacaceae				
*Portulaca oleracea* L. (Mattia 119)	Pucchiacchella Erva vasciulella	Aerial parts	Food: eaten in salads.	0.628
Primulaceae				
*Cyclamen purpurascens* Mill. (Mattia 018)	Piscialletto	Whole plant	Med.: it was put under the pillow of a baby who urinate in bed.	0.142
Punicaceae				
*Punica granatum* L. (Mattia 057)	Granata	Fruits	Med.: boiled, it was applied to aching breasts during the nursing	0.142
		Bark	Med.: a decoction is drunk internally as an abortive.	0.057
Ranunculaceae				
*Clematis vitalba* L. (Mattia 023)	Vitaglia	Young buds	Food: cooked, they are eaten in salads and with eggs in omelets.	0.314
		Branches	Dom.: to make baskets called “spaselle” where figs are dried.	0.414
Rosaceae				
*Crataegus monogyna* Jacq. (Mattia 070)	Calavrice	Flowers and leaves	Med.: an infusion is used as a sedative.	0.257
			Med.: an infusion is administered in treatment of stomachache.	0.271
			Med.: a decoction is used as a febrifuge.	0.157
			Med.: used in the preparation of the decoction called “o’ ricotto”.	0.514
		Branches	Dom.: to make sticks for agriculture tools.	0.371
*Prunus avium* (L.) L. (Mattia 062)	Ceraso	Fruits	Med.: the juice is considered a laxative.	0.271
		Stalk	Med.: a decoction is used for gargles in sore throat.	0.271
			Med.: a decoction is used as a diuretic.	0.142
			Med.: a decoction with seeds is used as an antitussive.	0.428
*Rosa canina* L. (Mattia 025)	Rosella; rosella sarvatica	Rosehips and leaves	Med.: an infusion is used in case of flu.	0.157
		Rosehips and flowers	Med.: used in the preparation of the decoction called “o’ ricotto”.	0.557
*Rubus caesius* L. (Mattia 033)	Rusto	Tender tops	Food: they are eaten with eggs in omelets.	0.271
		Fruits and Leaves	Med.: a decoction is used as an antidiarrheal.	0.285
*Sanguisorba officinalis* L. (Mattia 059)	Pane ‘e noce	Leaves	Med.: an infusion is claimed to be a gastric antispasmodic.	0.328
			Food: eaten in salads or cooked in preparation of “minestra maritata”.	0.514
*Sorbus domestica* L. (Mattia 088)	Sovere	Leaves	Med.: a decoction is used as an astringent.	0.342
			Med.: water where leaves are boiled is used topically on chilblain.	0.142
Rubiaceae				
*Galium verum* L. (Mattia 024)	Evera rà sbaria	Aerial parts	Med.: a decoction is used as a febrifuge.	0.285
Rutaceae				
*Ruta graveolens* L. (Mattia 039)	A’ruta	Leaves	Med.: fried in oil they are used for anti-inflammatory massages.	0.457
			Med.: the oil macerate is used as an anti-inflammatory for joints.	0.457
			Med.: an olive oil macerate is applied topically as an eye anti-inflammatory.	0.214
			Med.: used in the preparation of the decoction called “o’ ricotto”.	0.557
*Citrus limon* (L.) Burm. f. Osbeck (Mattia 086)	Limone	Fruits	Med.: the fresh juice is drunk in case of headache.	0.514
			Med.: one spoon of juice is employed in case of halitosis.	0.714
Salicaceae				
*Populus tremula* L. (Mattia 050)	Chiuppo	Bark	Med.: a water macerate is applied on warts.	0.271
			Med.: an infusion with leaves is claimed to improve memory.	0.114
*Salix alba* L. (Mattia 028)	Salece	Leaves	Med.: an infusion is used as a febrifuge.	0.328
		Branches	Dom.: called “turtielli”, they are is used to tie *Vitis vinifera* and to make baskets (“spaselle”).	0.314
*Salix purpurea* L. (Mattia 027)	Vitelle	Leaves	Med.: an infusion is used as a febrifuge.	0.328
		Branches	Dom.: called “turtielli”, they are is used to tie *Vitis vinifera* and to make baskets (“spaselle”).	0.314
Scrofulariaceae				
*Cymbalaria muralis* Gaertn., B. Mey., and Scherb. (Mattia 083)	Pratella sciurite	Aerial parts	Med.: a decoction is used as a cicatrizer for wounds.	0.371
Solanaceae				
*Capsicum annuum* L. (Mattia 058)	Pupaino	Fruits	Med.: an olive oil macerate is used for anti-rheumatic massages.	0.585
*Datura stramonium* L. (Mattia 076)	Fetiente	Leaves	Med.: smoked as an anti-asthmatic.	0.214
*Lycopersicon esculentum* Mill. (Mattia 017)	Pummarola	Fruits	Med.: applied on insect bites as a decongestant.	0.485
		Aerial parts	Med.: an infusion with *Rosa canina* leaves is claimed to be useful in treatment of kidney stones.	0.142
*Nicotiana tabacum* L. (Mattia 001)	Erbasanta	Fresh leaves	Med.: applied against toothache.	0.614
*Solanum melongena* L. (Mattia 016)	Mulegnana	Leaves	Med.: boiled and applied on hemorrhoids as an anti-inflammatory.	0.271
*Solanum tuberosum* L. (Mattia 008)	Patana	Tuber	Med.: crushed, it is applied on burns as a lenitive.	0.714
			Med.: it is cut in half and put on the forehead to relieve headache.	0.400
Tiliaceae				
*Tilia platyphyllos* Scop. (Mattia 115)	Teglia	Flowers	Med.: a decoction is used as a sedative.	0.357
			Med.: a decoction with *Ruta graveolens* and *Eucalyptus globulus* leaves is used as a febrifuge.	0.371
			Med.: used in the preparation of the decoction called “o’ ricotto”.	0.557
		Bark	Med.: a decoction is used in treatment of cystitis.	0.271
			Med.: a water macerate is used as a lenitive for burns.	0.200
Umbelliferae				
*Angelica sylvestris* L*.* (Mattia 195)		Leaves	Med.: a decoction is considered a vermifuge.	0.142
*Apium graveolens* L*.* (Mattia 068)	Accio	Leaves	Med: an infusion with *Parietaria officinalis* aerial parts and *Petroselinum sativum* roots is claimed to be effective in treatment of kidney stones.	0.185
*Daucus carota* L. (Mattia 053)	Pastinaca	Root	Med.: eaten as a diuretic.	0.214
			Med.: eaten as a laxative.	0.314
		Flowers	Dom.: used to obtain a dye for paintings.	0.085
*Foeniculum vulgare* Mill. (Mattia 014)	Finucchiello	Fresh leaves	Med.: a decoction with *Matricaria chamomilla* heads is used in case of headache.	0.385
			Med.: an infusion is used as a carminative.	0.414
		Fruits	Med.: fruits are smoked against toothache.	0.057
			Food: used for liqueurs and to aromatize foods.	0.714
*Petroselinum sativum* Hoffm. (Mattia 002)	Petrusino	Fresh leaves	Food: used to aromatize foods.	0.785
		Roots	Med: an infusion with *Parietaria officinalis* aerial parts and *Apium graveolens* leaves is claimed to be effective in treatment of kidney stones.	0.228
Urticaceae				
*Parietaria officinalis* L. (Mattia 037)	Paredara	Aerial parts	Med.: a decoction with *Matricaria chamomilla* heads is used against peripheral edemas. A with white egg a wrap is prepared for contusion and/or distortion.	0.228
			Med.: a wrap prepared with an albumen is used as a decongestant to treat contusions and/or distortions.	0.585
			Med.: an infusion with *Petroselinum sativum* roots and *Apium graveolens* leaves is claimed to be effective in treatment of kidney stones.	0.228
			Med.: used in the preparation of the decoction called “o’ ricotto”.	0.557
			Dom.: a mix of sand, water and *P. officinalis* is used to clean wine stains from carboys and bottles.	0.371
*Urtica dioica* L. (Mattia 085)	Ardica	Aerial parts	Med.: a decoction is used as an expectorant, sometimes adding barks of *Vitis vinifera* and leaves and roots of *Malva sylvestris.*	0.342
			Med.: a decoction is used as a depurative.	0.414
			Vet.: a decoction is administered to animals to expel afterbirth.	0.114
			Food: boiled, are eaten in salads or with pasta.	0.128
			Feed: used as feed for cows.	0.285
			Dom.: macerated for 15 days and sprayed on vegetables to protect them from insects.	0.214
*Urtica urens* L. (Mattia 074)	Ardica	Aerial parts	Med.: a decoction is used as an expectorant, sometimes adding barks of *Vitis vinifera* and leaves and roots of *Malva sylvestris*.	0.342
			Med.: a decoction is used as a depurative.	0.414
			Vet.: a decoction is administered to animals to expel afterbirth.	0.114
			Food: boiled, are eaten in salads or with pasta.	0.128
			Feed: used as feed for cows.	0.285
			Dom.: macerated for 15 days and sprayed on vegetables to protect them from insects.	0.214
Valerianaceae				
*Centranthus ruber* (L.) DC. (Mattia 174)	Cannaviello; valerianella rossa	Whole plant	Med.: a decoction is used as a mild sedative.	0.242
Verbenaceae				
*Lippia triphylla* (L’Hér.) Kuntze (Mattia 109)	Erba cedro	Leaves	Med.: an infusion is claimed to be a digestive.	0.514
			Med.: a decoction is used as a mild sedative.	0.271
			Med.: used in the preparation of the decoction called “o’ ricotto”.	0.557
Violaceae				
*Viola odorata* L. (Mattia 141)	Violetta	Roots	Med.: a decoction with *Malva sylvestris* leaves and *Salvia officinalis* aerial parts is considered an antitussive.	0.414
Vitaceae				
*Vitis vinifera* L. (Mattia 026)	Vite	Fruits	Med.: dried grapes were eaten in the case of flu.	0.342
			Med.: the marc is used topically in treatment of arthritis.	0.271
		Bark	Med.: a decoction with *Malva sylvestris* leaves is used against bronchitis.	0.414

UV use value, *Med* plant used in human medicine, *Vet* plant used in veterinary medicine, *Food* plant used as human food, *Feed* plant used as animal feed, *Dom* Plant used for domestic use

The results of the present work have been compared to ethnobotanical data from nearby zones of Southern Italy.

### Human medicine

The plants, used to cure human ailments, have been categorized into 11 categories; consequently, a single species could be listed in several illness categories (Table 2). Among these plants the highest number is recorded for UG (about 15%) and GI (about 14%) groups. Less frequently, plant species are used for OR, ENT and OP (about 2%).

**Table 2:** Plants used in human medicine

Illness categories	Number of species	Number of uses	Percentage
Urogenital system (UG)	24	30	15
Gastrointestinal tract (GI)	27	28	14
Systemic disorders (SY)	23	24	12
Skin diseases (SK)	23	24	12
Anti-inflammatory (ANT)	14	18	9
Respiratory system diseases (R)	12	12	6
Neuropsychiatric diseases (NP)	8	8	4
Cardiovascular diseases (CV)	5	6	3
Oral cavity diseases (OR)	4	4	2
Ear, nose and throat diseases (ENT)	4	4	2
Ophthalmologic diseases (OP)	3	3	2

One hundred and one species, belonging to 48 families, were reported for the human uses. The most cited families were Lamiaceae (11 species), Asteraceae (8 species), Rosaceae and Solanaceae (6 species).

In particular, the decoction of rhizome of *Arundo donax* L. was employed against gastric affections, use reported also by De Feo and coworkers [5], De Feo and Senatore [13], and Guarrera and Savo [17]. Also, a decoction of *Lavandula angustifolia* Mill. has a similar use.

For systematic diseases, we reported the application of flowers of *Spartium junceum* L. and, in particular, for the treatment of diabetes, we cited the application of fresh leaves of *Artemisia absinthium* L.

In the same Asteraceae family, *Cichorium intybus* L. and *Cynara scolymus* L. were reported for liver pathologies; *Bellis perennis* L. heads, together with *Mercurialis annua* L. (Euphorbiaceae), were employed as a febrifuge. In literature, other authors [5, 6, 12, 16, 17, 21, 22, 26] cited depurative and laxative uses of *C. intybus*.

Some species are known for their diuretic activity: the decoctions of the leaves of *Borago officinalis* L., *Ocimum basilicum* L., *Asparagus acutifolius* L, *Morus alba* L., *Morus nigra* L., *Zea mays* L., *Prunus avium* L. are employed for this purpose. Pieroni and coworkers [26], Savo and coworkers [4], and Scherrer and coworkers [1] cited the decoction of aerial parts of *B. officinalis* as a depurative.

Bark of *Punica granatum* L. is used in a preparation of an abortive decoction; this use seems to be new in the Italian ethnobotanical literature.

*Cynodon dactylon* (L.) Pers. and *Sambucus nigra* L. are utilized to cure female infertility. A rhizome decoction of *C. dactylon* is known for its application in renal stones, as an urinary anti-inflammatory [4, 5, 12, 14, 16, 19]. The plant is also reported to cure inflammations of the digestive and genital–urinary apparatuses (diuretic, “refreshing,” renal colics) [4, 22, 25]. *Cyclamen purpurascens* Mill. is put under the pillow of babies who urinate in bed.

Twenty-three species are cited for their use in skin pathologies: in particular, we can highlight the use of gel from the stems of *Opuntia ficus-indica* Mill. as a lenitive for skin [21], a water macerate from bark of *Tilia platyphyllos* Scop. used on burns, the leaf oil macerate of *Ruta graveolens* L. as a skin anti-inflammatory and for the treatment of ophthalmic affections. De Feo and coworkers [5, 12] referred the use of *O. ficus indica* as a plaster: in particular, the powdered branches are used to treat corns and frostbite.

*Salvia officinalis* L. is directly applied on skin affected by *Herpes zoster*. The decoctions of leaves of *Althaea cannabina* L. and *Cymbalaria muralis* G. Gaertn., B. Mey., and Scherb. are applied externally to have an anti-inflammatory action.

The rhizome of *Arum italicum* L. is used as a skin decongestant: a similar use is reported by other Authors [6, 29]. Instead, Guarrera [16] and Montesano and coworkers [22] cited the topical applications of sap as healing of warts.

Of importance, the use of *Cannabis sativa* L. in medicine and for domestic uses: this species was widely cultivated in past time for the production of textiles and twines; today, its cultivation is totally fallen into disuse, due to the introduction of synthetic fibers.

A wrap of *Vincetoxicum hirundinaria* Medik. is used against contusions and distortions; the leaves of *Hedera helix* L. are boiled until to be a gel which can be applied as anti-rheumatics [5, 21]; an infusion of the leaves is reported as an anti-neuralgic.

Two ways of administration of *Matricaria chamomilla* L. should be cited: an infusion of its flower heads with *Laurus nobilis* L. leaves for the treatment of edemas; a poultice of the plant, applied externally, against hematomas and traumas. These plants were reported in literature with the same uses [1, 4, 5, 12, 21, 25]. It is of interest that a decoction of flowering heads of the first plant, mixed with mallow (*Malva sylvestris* L.) flowers, can be used to soothe the cough.

A decoction with *L. nobilis* is reported against cough or belly pains, also used for goats [1]. An infusion of *M. chamomilla* and *Lactuca sativa* L. is considered an intestinal spasmolytic.

A decoction of the plant, pure or with *M. sylvestris* is claimed useful against cough and bronchitis [21], alone or with chamomile for digestive purpose. Moreover, a decoction of its aerial parts is reported as a mild laxative [12, 17, 22] and as a gastric antispasmodic [14].

The same parts of this plant are used for their sedative action; a similar action is possessed by an infusion of flowers of *Lavandula angustifolia* and *Papaver rhoeas* L. The same or similar use for poppy is reported by other Authors [4, 5, 12, 16, 17, 21, 26]. Di Novella and coworkers [14] cited the use of the poppy as an hypnotic.

Cigarettes made of leaves of *Datura stramonium* L. are used as an anti-asthmatic; this use is reported in literature [5, 14, 16, 21]. Some species of *Thymus* and *Urtica* are utilized as an expectorant also with *Ceterach officinarum* DC [12]; a decoction of *Vitis vinifera* L. is used with *M. sylvestris* leaves against bronchitis and a decoction of *Origanum vulgare* L. is used against upper respiratory affections. Menale and coworkers [21] reported the use of oregano and *M. sylvestris* in case of cough. Guarrera [16] indicated the use of some species of *Thymus* in case of colds.

*Ceterach officinarum* DC. is known with the popular name of “spaccapietre” (stone-breaker) due to its use, mainly in Basilicata and Puglia regions, in kidney lithiasis [14, 16].

In plants acting on cardiovascular system, *Calystegia sepium* L. is used to decrease blood pressure; this use was reported in Italian ethnobotanical literature [5]. The fruits and leaves of *Olea europea* L. are utilized for the same hypotensive effect; this use was already reported [5, 12, 16, 21, 25].

The seeds of *Foeniculum vulgare* Miller are smoked against toothache; this use seems to be peculiar of the studied area.

The fresh leaves of *Vincetoxicum hirundinaria* are used as a gargle for the same pain. Further, the plant is cited as ingredient of “ricotto” (a remedy used as panacea: for the explanation, see below).

Leaves of *Quercus ilex* L. are employed in decoction with *Urtica urens* L. for gargles against throat inflammations.

We can cite the employment of fresh leaves of *Foeniculum vulgare* for headache. An infusion of *Diplotaxis tenuifolia* L. is reported as a male aphrodisiac [4, 5, 16, 17, 21].

An infusion of flowers and leaves of *Polygonum aviculare* L. is used as an appetite stimulant for children.

Some preparations are based on mixtures of multiple plants, as reported in Table 3: in particular, these preparations are used for edemas, for kidney stones, and, above all, for respiratory diseases; *M. sylvestris* and *M. chamomilla* are most common plants in these multiple preparations.

**Table 3:** Some preparations based on mixtures of multiple plants

1	Poultice	*Matricaria chamomilla*	For edemas
		*Laurus nobilis*	
2	Poultice	*Sambucus nigra*	Leg edema
		*Parietaria officinalis*	
		*Vincetoxicum hirundinaria*	
3	Decoction	*Parietaria officinalis*	Peripheral edemas
		*Matricaria chamomilla*	
4	Infusion	*Parietaria officinalis*	Kidney stones
		*Petroselinum sativum*	
		*Apium graveolens*	
5	Infusion	*Lycopersicon esculentum*	Kidney stones
		*Rosa canina*	
6	Vapor inhalation	*Eucalyptus globulus*	Sinusitis
		*Urtica dioica*	
		*Cynodon dctylon*	
		*Parietariai officinalis*	
		*Citrus limon*	
7	Vapor inhalation	*Laurus nobilis*	Sinusitis
		*Borago officinalis*	
		*Populus tremula*	
		*Myrtus communis*	
		*Urtica dioica*	
8	Decoction	*Ficus carica*	Antitussive
		*Malus domestica*	
	The same decoction also with	*Juglans regia*	Antiutussive
		*Malva sylvestris*	
		*Matricaria chamomilla*	
9	Decoction	*Viola odorata*	Antitussive
		*Malva sylvestris*	
		*Salvia officinalis*	
10	Decoction	*Urtica* species	Expectorant
		*Vitis vinifera*	
		*Malva sylvestris*	
11	Decoction	*Vitis vinifera*	Bronchitis
		*Malva sylvestris*	
12	Decoction	*Cynodon dactylon*	Urinary anti-inflammatory and diuretic
		*Urtica dioica*	
13		*Tilia platyphyllos*	Febrifuge
		*Ruta graveolens*	
		*Eucalyptus globulus*	

In all investigated zones, the use of a decoction of some plant species, locally named “o’ ricotto,” is very diffused, mainly among the elderly. This remedy is used as a panacea to cure numerous diseases, as abdominal pains or colds. It has a very good taste, so, in many cases it is drunk with pleasure. Many interviewees give this type of preparation to ill children. In each locality, there are some people which, during spring and summer, care of collect and dry the plants to prepare this decoction.

The list of the species used for this decoction is shown below, with employed parts, taking into the consideration that each people modifies the recipe to his liking. Twenty-nine plants (reported in Table 4) were used, belonging to 18 families: Labiatae (9 species), Compositae (3 species), and Rosaceae (2 species) as the most represented.

**Table 4:** The list of the species used for “ricotto” decoction

*Achillea millefolium* L*.*	Leaves and flowers
*Balsamita major* L.	Leaves and flowers
*Crataegus monogyna* Jacq.	Flowers and leaves
*Cynodon dactylon* Pers.	Roots
*Ficus carica* L.	Leaves, dried syconia
*Fraxinus ornus* L.	Leaves
*Hypericum perfoliatum* L.	Aerial parts
*Juglans regia* L.	Pericarp
*Lavandula officinalis* L.	Flowering tops
*Laurus nobilis* L.	Leaves
*Lippa triphylla* O. Kuntze	Leaves
*Malva sylvestris* L.	Flowers and/or root
*Matricaria chamomilla* L.	Flowering fields
*Mentha spicata* L., *Mentha rotundifolia* (L.) Hudson, Mentha*piperita L.	Flowers and leaves
*Myrtus communis* L.	Flowers and leaves
*Nepeta cataria* L.	Flowers and leaves
*Ocimum basilicum* L.	Leaves
*Ostrya carpinifolia* L.	Leaves
*Parietaria officinalis* L.	Leaves
*Polygonum aviculare* L.	
*Rosa canina* L.	Flowers and/or fruits
*Rosmarinus officinalis* L.	Aerial parts
*Ruta graveolens* L.	Leaves
*Salvia officinalis* L.	Flowers and leaves
*Sambucus nigra* L.	Flowers
*Thymus vulgaris* L.	Flowers and leaves
*Tilia platyphyllos* Scop.	Flowers

### Veterinary medicine and feed

Eight percent of the reported species are employed for veterinary uses or as animal feed. Among the four species reported for veterinary use, the macerated oil of *Allium sativum* L. is employed against chicken diseases. Normally, the use of *Fraxinus ornus* L. is very diffused for a high number of human pathologies [4, 16]; instead, we cite its veterinary use: an aqueous macerated of the plant is employed to cure cooling diseases of gallinaceans (local name “pepitola”). Also in Cilento area, a decoction of trunk barks and young branches of the plant was administered to young chicks as a gastric disinfectant [14].

*Urtica dioica* L. and *U. urens* L. are used for cattles to facilitate placental disposal; moreover, these plants are used as a feed.

Other six species reported were employed as a feed: in an age in which synthetic foods often replace natural fodders, it is worth remembering some foods of plant origin traditionally given to domestic animals. Among the new uses, we report *Cnicus benedictus* L. as feed for donkeys and *Triticum turgidum* L. as a beverages for animals: in particular, dirty dishes are washed with seed bran in hot water and therefore, this water is given to drink to the pets. It is claimed that species used as animal feed improve animal health, as well as the quality of milk and dairy products.

### Human food and food aromatizer

Wild foods constitute an essential component of people’s diets around the world [11]. In general, dishes made with wild plants are often identified as functional foods (foods with biological effects that go beyond their mere nutritional properties) and wild plants can contribute to overcoming periods of food or income shortages [11].

Thirty-six species (30%) are employed as food plants in the studied area. The plants are either eaten raw, mixed with other vegetables or in salads, when they are prepared with young and tender leaves that when picked in the early vegetative stage of the rosetta have a less bitter taste, or boiled, when harvested as older leaves, even in mixed vegetable soups [28]. The recipe of “Minestra maritata,” prepared during Easter time, is reported in Table 5: specifically, eight of these plants are Compositae, two are Cruciferae, two are Plantaginaceae, and one is of Rosaceae family.

**Table 5:** The list of the species used for “Minestra maritata”

*Silybum marianum* (L.) Gaertn.	
*Chondrilla juncea* L.	
*Cichorium intybus* L.	
*Crepis vesicaria* L.	
*Helminthotheca echioides* (L.) Holub	
*Sonchus oleraceus* (L.) L.	
*Sanguisorba officinalis* L.	
*Taraxacum campylodes* G.E.Haglund	
*Capsella bursa-pastoris* (L.) Medicus	
*Reichardia picroides* (L.) Roth.	
*Nasturtium officinale* R. Br.	
*Plantago lanceolata* L.	
*Plantago major* L.	

Also Guarrera and Savo [18] cited this traditional soup of Campania region made by *Cichorium intybus*, *Foeniculum vulgare*, *Reichardia picroides*, *Sonchus asper*, cabbage (*Brassica oleracea*), celery (*Apium graveolens* L.), endive (*Cichorium endivia* L.), lettuce (*Lactuca sativa* L.), onion (*Allium cepa* L.). *S. marianum* was eaten as a snack also in Basilicata region [6, 18, 22]; moreover, the plant is eaten in salad in some Italian regions [16, 24]. *C. juncea*, *C. intybus*, *C. vesicaria*, *S. oleraceus*, and *S. officinalis* were cited by Guarrera and Savo [17, 18] as nutraceuticals.

*C. intybus*, *Crepis bursifolia* L., *Crepis leontodonotides* All., *Sonchus asper* (L.) Hill, and *S. oleraceus* are reported by Di Novella and coworkers [14] as some of the main ingredients of the “minestra terrana,” a very common soup made by 12 wild species. The ingredients are boiled in water and they are mixed with olive oil, *Allium sativum*, and other condiments.

*Foeniculum vulgare* L. is employed as a food and for the preparation of liquors. Also some species of Asteraceae are used for preparation of liquors or as food in different kinds of “minestra.” So, in literature, the leaves of *C. intybus* are reported added to soups, eaten as salad or fried, and finally as an ingredient of “minestra” [1, 6, 24]. *S. nigra* leaves are eaten cooked with eggs, while its fruits are employed in typical marmalades. Some species of *Mentha* are used to aromatize a typical food made of veal and pork spleen. *Ceratonia siliqua* L. is used as a food for children.

The leaves of *Armoracia rusticana* P. Gaertn., B. Mey., and Scherb., together with *Anethum graveolens* L. and *Laurus* leaves, were used as flavoring agents for pickled fruits of *Lycopersicon*, with water, vinegar, salt, and sugar; the remaining solution of the tomato pickling process is drunk.

Furthermore, two typical liqueours, “nocito” or “nocillo” (made with *Juglans regia* hull) and “cient’erb” (a complex mixture of plants), are prepared: the plants, collected in St. John’s day, are macerated until the Assumption day, when the mixture is filtered and sugar is added.

### Domestic and handicraft uses

In the studied area, a considerable number of plants (24%) are employed for domestic uses or in local handicrafts: Fagaceae (3 species), Urticacae (3 species), Asteraceae, Lamiaceae, and Salicaceae (2 species) are the most represented families. *Cannabis sativa* L. was used in the manufacturing of cordages, a key factor for city economy: Di Novella and coworkers [14] reported the stems of the plant used to obtain textile fibers. Moreover, the fibers of *C sativa* mixed with eggs were used to make anti-inflammatory bandages.

**Fig. 2:**
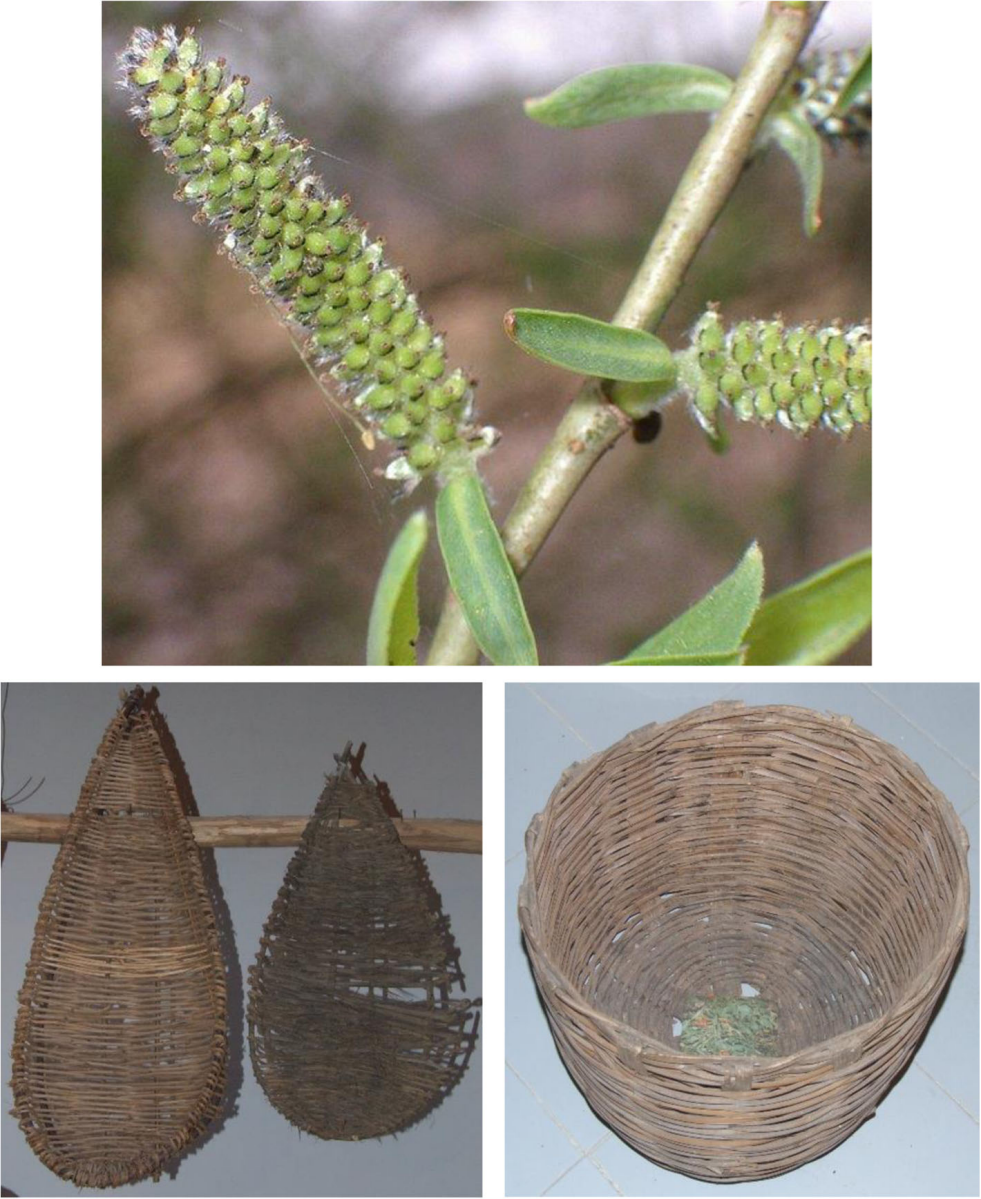
*Salix alba* and *S. purpurea* young branches used to manufacture baskets

*Daucus carota* L. is reported for its use in color for paintings. We can cite the particular use of *Vincetoxicum hirundinaria*: a water maceration of this plant with *Urtica urens* leaves is sprayed on the vegetables to send away insects. The women used, to wash themselves, perfumed water obtained from the maceration of fresh flowers of *Bellis perennis* L. or inflorescences of *Lavandula angustifolia* Miller. Some plants are reported for their handmade products: the wood of *Acer campestre* L. is employed to make tool handles, toys, and a traditional typical musical instrument known as “ciaramella.” *Arundo donax* L. is used to do baskets, musical instruments, and as a support for vegetables; a similar use is reported by Di Novella and coworkers [14] and Passalacqua and coworkers [24]. *Salix purpurea* L. and *S. alba* L. are used to tie grape plants [16, 22] and to manufacture baskets (Fig. 2) [14].

In past times, *Quercus* species were employed to make vats, barrels, and generally tools; moreover, a diffused utilization of *Castanea sativa* Mill. is the construction of different shape and size barrels.

*Polygonum aviculare* (whole plant) is boiled to wash barrels with *Foeniculum vulgare*, *Laurus nobilis*, *Nepeta cataria*, and *Citrus limon* leaves.

The leaves of *Armoracia rusticana* are smoked; the leaves of *Saponaria officinalis* L. are used to clean the hands, especially after the production of tobacco from *Nicotiana tabacum* plant. This use is diffused also in other areas [14, 24].

A mix with sand, water, and *Parietaria officinalis* is used to clean wine stains from carboys and bottles; the same use is reported in literature [14, 16, 24].

Out of the ordinary is the use of *Euphorbia dendroides* L.: a water macerate is sprayed on fruit-trees to prevent theft [13]. *Ceratonia siliqua* seeds were used to make necklaces.

### Taxonomic diversity, plant parts used, and modes of consumption

**Fig. 3:**
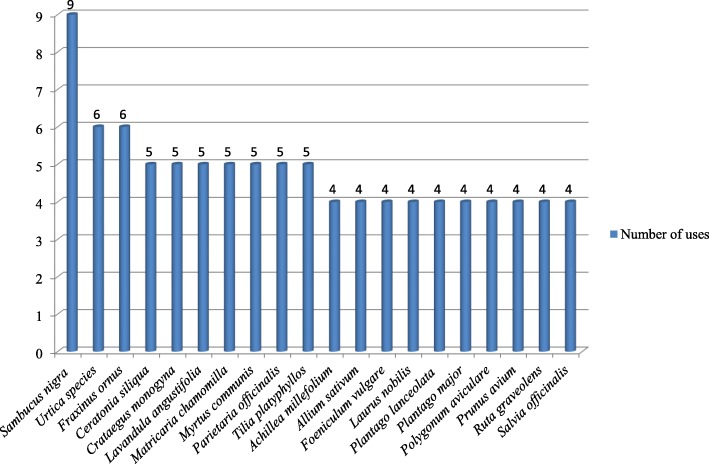
The species most cited in the study

**Fig. 4:**
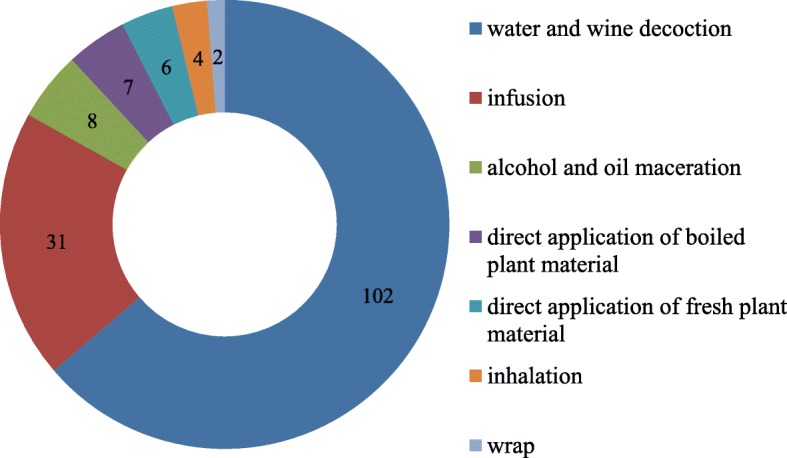
Different preparations and application processes of medicinal plants

**Fig. 5:**
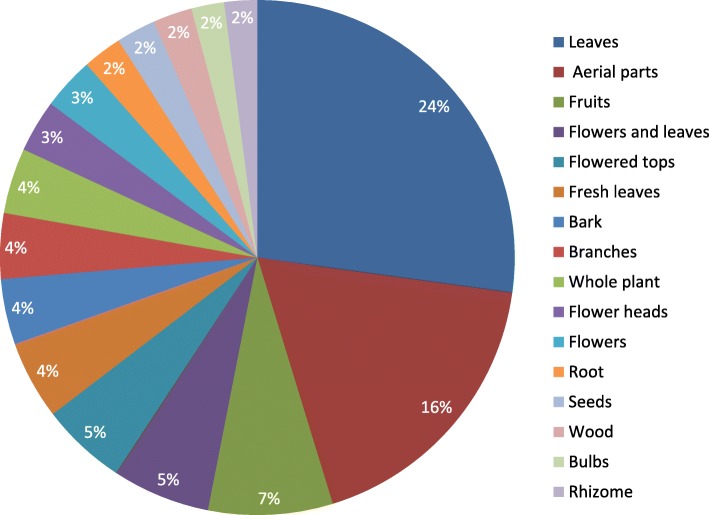
The main plant parts used in preparation an application processes

The species most cited in the study are reported in Fig. 3. Different preparations and application processes of medicinal plants used are as reported in Fig. 4. For plants not with medicinal uses, we registered two decoction preparations, eight macerate preparations, and three preparations with boiled plants. Overall, decoction and infusion are the most cited preparations. The majority of remedies were prepared from dried material. In some of cases (21), the plants are used in the fresh state. The plant parts used for these types of medical preparations are, above all, leaves (66 cases, mean UV value 0.38), aerial parts (44 cases, mean UV value 0.36), flowers, flowering tops, flowering heads (in total, 30 cases, mean UV value 0.35), fruits (19 cases, mean UV value 0.40), and barks (10 cases, mean UV value 0.29). The main parts used are reported in Fig. 5. The dosage is empirical: generally, for 1 L of water, two handfuls of plant were added.

### General considerations

The knowledge about medicinal plants and other useful ones is still alive, passed down from generation to generation; however, people over 50 years old has retained this kind of information.

In the investigated area, healers are still respected: some of these persons follow these practices and are proud to be the last guardians of a now lost culture; sometimes they report that they have cured some people in cases where the official medicine has failed.

Several species are harvested at dawn on St. John’s Day (June 24). The eve of Assunta day, August 14, is another important day for the collection of specific plants, known as “erve ra ‘Maronna” (herbs of Santa Maria). In popular belief, the therapeutic features of these plants are higher if these species are collected during waning moon, in a period named locally “a’ mancanza” (meaning falling moon). The plants are cleaned and divided in small pieces, mixed each other in different quantities for species, shade dried.

Data analysis showed that the people that use traditional remedies possess the knowledge of a high number of plants. This can reflect the transmission of the phytotherapeutical knowledge among the investigation area. Generally, women are depositaries of the medicamental properties of plants, also because from ancient time the female line takes care of lands dedicated to gardens and cultivation of cereals, while the male line is dedicated to pastoral activities.

Furthermore, for most plant species, knowledge appears to be homogeneous, very scarce, or unaffected by external factors. Their effectiveness can sometimes be justified not only by the known presence of active chemical substances, but also by the widespread practice and even by the observation of the concrete benefit obtained by the informer. The use of different species in different Italian areas often depends on the local availability of plants or the presence of typical species: in the literature, it emerges that some wild plants have a very limited use. Since time immemorial, plants have been the first medicines to cure diseases. Man becomes aware of the ethnobotanical application of plants through trial and error. This knowledge has been transmitted orally from generation to generation and has been applied in different parts of the world [32]. Furthermore, ethnobotanical research discovers plant resources that can be used to obtain new compounds that lead to the development of innovative drugs for the treatment of diseases [33, 34]: in fact, the discovery of new botanical drugs and new food crops depends on ethnobotanical knowledge [35]. Finally, the ethnobotanical study of medicinal plants is based on the acknowledgment of contributions made by local communities and/or by single persons who share specialized acquaintance; on the other hand, it can contribute to help native people and the preservation of biodiversity in their environments [36].

Ethnopharmacology is based on the recognition that people, throughout history, have utilized natural products as therapeutic agents and traditional medicinal knowledge can be used as a tool to obtain more information about the therapeutic capabilities of a natural product [37]. Traditional understanding is a resource that has been below estimated in the past, and the actual contribution of ethnopharmacology to drug finding has often been discontinuous.

The aim is to move forward, mainly in the context of the sources available nowadays, formalizing the use of ethnopharmacology to increase the development of drug discovery and quicken the recognition of novel therapeutics [37].

Generally, nowadays, in veterinary medicine, traditional natural remedies are substituted by synthetic pharmaceuticals for the cure of animals. In the present time, official veterinary practices take care of animal health from all point of view and affect most of the veterinary procedures realized by shepherds and farmers. However, in various areas of the Mediterranean region, such folk practices resist and natural ethnoveterinary remedies are now only rarely employed by people; the reason why these remedies are referred by few informants [27].

Some plants, different from the mixtures of herbs that are randomly collected in the field, are used as animal fodder, to maintain their good health conditions. Generally, fodder plants were picked by women near to the village, but sometimes they were mowed and piled by men in front of their house.

Some botanical foods have been cited and mentioned in several areas, showing that there is an ethnobotanical convergence between the various Italian regions [14, 15, 17, 18]. “Let food be your medicine”: the Hippocratic declaration was linked to the traditional idea of food and reflects the approach of the Greek physician to medicine, highlighting the meaning of diet and existing habits in preserving health from diseases. In fact, in ancient time, many plant species employed in the medical practice were also consumed as aliments [28]. Several plants are consumed by people because they help maintain health. These plants may have a specific use or multiple properties and are able to counter and prevent a wide set of medical conditions [17]. Edible plants should be considered for their important socio-cultural, health, and economic benefits for both local communities and farmers engaged in their production and harvesting [11].

Dietary patterns change rapidly all over the world. The local food knowledge available, which forms the basis of many local traditions, is drastically diminishing.. At the same time, consumers demand novel types of tasty food, which is easy to prepare. In the Mediterranean, vegetables and salads, made from wild greens, have been particularly important as local (traditional) foods since ancient times. In recent years, wild food plants have increasingly became the focus of attention for many ethnobotanists in Europe. There are several reasons for this: the renewed interest in local traditional foods and in plant food sources; the related concepts of *terroir* and intangible cultural heritage, and the potential of these foods as nutraceuticals, in the prevention of diseases [15] and in the contribution to a healthy and balanced diet [23].

The rediscovery of the folk uses of plants in the area under consideration is not only of historical and scientific value, but could also represent a future, economic potential for the area. Several plants could still today be involved in the production of typical and appealing artifacts. In particular, the production of typical objects that are now on the decline (collars, baskets, clothes of particular textile fibers, and generally the artifacts under sale) could regain importance in the local economy [38].

## Conclusions

The documentation of 119 traditional medicinal plants and preparations such as “ricotto” indicates that knowledge of popular plants in the Cava de’ Tirreni area still exists and that wild plants are now used by people in their daily lives. Unfortunately, the traditional use of plants is declining and the according knowledge is mainly restricted to the elderly.

Moreover, the comparison of the documented species and their uses with ethnobotanical literature of other Italian regions reveals that the traditional plant knowledge in this area shows strong similarities with adjacent Southern Italian areas. Some of the recorded species and administration processes however seem to be unique for the zone.

## Supplementary information

**Additional file 1.** Questionnaire form for ethnobotanical research.

## Data Availability

Raw data can be requested from the corresponding author.
